# Optional Revascularization Strategies for Patients with ST-Segment Elevation Myocardial Infarction and Multivessel Disease

**DOI:** 10.31083/j.rcm2506209

**Published:** 2024-06-04

**Authors:** Peng-Yu Zhong, Bin Sun, Hai-Ping Cao, Wei Wang, Ting-Lin Xiong

**Affiliations:** ^1^Department of Cardiology, Nanchong Central Hospital (Nanchong Hospital of Beijing Anzhen Hospital/The Second Clinical College of North Sichuan Medical College), 637000 Nanchong, Sichuan, China

**Keywords:** culprit-only revascularization, complete revascularization, multivessel disease, ST-segment elevation myocardial infarction

## Abstract

Percutaneous coronary intervention is the main strategy of revascularization and has been shown to improve outcomes in some patients with ST-segment elevation myocardial infarction (STEMI). However, multivessel disease (MVD), a common condition in these patients, is associated with worse clinical outcomes compared to single-vessel disease. Despite intervention being a standard treatment for coronary artery disease, optimal strategies and timings for patients with STEMI and MVD remain unclear. Numerous studies and meta-analyses have investigated this topic; however, many current conclusions are based on observational studies. Furthermore, clinical guidelines regarding the management of patients with STEMI and MVD contain conflicting recommendations. Therefore, we aimed to compile relevant studies and newly available evidence-based medicines to explore the most effective approach.

## 1. Introduction

ST-segment elevation myocardial infarction (STEMI) is an underlying disease in which approximately 50% of these patients present with multivessel lesions at the time of primary coronary angiography [1]. These patients have higher short- and long-term mortality rates compared to patients with less extensive coronary lesions [2, 3]. Percutaneous coronary intervention (PCI) is an effective technique, providing significant prognostic improvement and net clinical benefits for patients with STEMI.

Multiple randomized controlled trials have demonstrated that complete revascularization is associated with more clinical benefit than culprit-only revascularization (COR) in STEMI patients without cardiogenic shock [4, 5, 6, 7, 8]. Therefore, both guidelines in Europe and America suggest prioritizing treatment on the culprit vessel during the initial procedure while addressing the remaining lesions through subsequent revascularization for patients with STEMI and multivessel disease (MVD), as well as the absence of cardiogenic shock [9, 10]. On the contrary, immediate interventional treatment of non-culprit vessels at the primary PCI was previously considered a restricted zone in patients with STEMI and MVD. Indeed, earlier observational studies had shown worse outcomes when non-culprit-related artery revascularization was performed during primary PCI [11, 12]. However, optimizing stent design, applying novel antiplatelet agents, and developing intracavitary imaging has significantly driven the performance of immediate complete revascularization in patients with STEMI. Hence, it remains unclear whether performing complete revascularization benefits these patients. Furthermore, applying fractional flow reserve (FFR) can provide a more precise assessment and reduce the incidence of major adverse cardiac events (MACEs) after PCI [13]. However, it is unclear whether it can provide more clinical benefits to patients with STEMI and MVD. Finally, the contemporary development of mechanical support devices, such as extracorporeal membrane oxygenation and intra-aortic balloon pumps, has improved the performance of immediate complete revascularization in these patients with cardiogenic shock.

Although an increasing number of randomized controlled trials have testified that complete revascularization can bring more clinical benefits than COR, there remains a series of controversies. This study aims to report the related clinical research and the new progress in this topic.

## 2. Superiorities and Inferiorities of Complete Revascularization

**Fig. 1.**
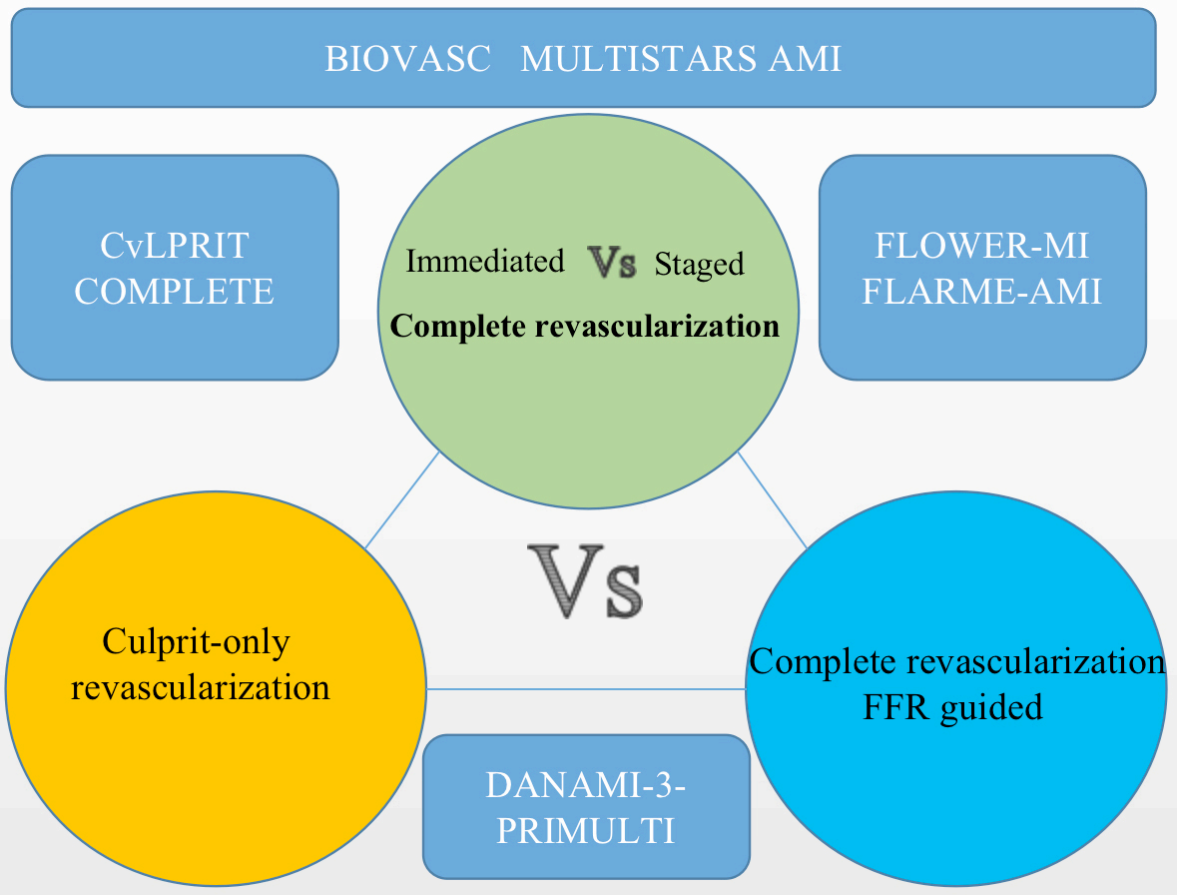
**The main large-sample randomized controlled trials comparing the four revascularization strategies.** FFR, fractional flow reserve.

A large number of randomized controlled trials have been conducted recently aimed at investigating the optimal revascularization regimen, and the related research topics and clinical trials in this field are presented below (Fig. 1). Anatomically, complete revascularization is defined as the successful treatment of all anatomically significant lesions during the index procedure, which can be emergency or elective surgery. These lesions typically have a stenosis diameter of ≥50% or ≥70%, with reference diameters of ≥1.5 mm or ≥2.0 mm [14]. Culprit vessel revascularization plays a crucial role in STEMI treatment, and numerous studies have confirmed that non-culprit revascularization is also essential and significantly improves clinical prognosis. The main disadvantages of complete revascularization are the increased contrast load, risk of complications, radiation exposure, and operation time. There are 11 non-randomized studies, which were compared to COR and complete revascularization, although none provided a unanimous conclusion (Table 1, Ref. [12, 15, 16, 17, 18, 19, 20, 21, 22, 23, 24]). On the contrary, most randomized controlled trials favor complete revascularization, including immediate and staged (Table 1, Ref. [4, 5, 25, 26, 27, 28]).

**Table 1.** **The published studies comparing complete revascularization with COR**.

Trials	Year	Sample	Patients	Main conclusions	Reference
Randomized controlled trials					
Mehta *et al*.	2019	4041	STEMI	Complete revascularization significantly reduced the risk of cardiovascular death, myocardial infarction, and ischemia-driven revascularization.	[4]
Hamza *et al*.	2016	100	STEMI	Complete revascularization is associated with lower risk of MACEs in diabetic patients with MVD.	[27]
Gershlick *et al*.	2015	296	STEMI	Immediate complete revascularization significantly reduced the risk of composite primary outcomes.	[25]
Wald *et al*.	2013	465	STEMI	Multi-vessel PCI significantly reduced the risk of adverse cardiovascular events.	[5]
Dambrink *et al*.	2010	121	STEMI	No differences were found.	[26]
Di Mario *et al*.	2004	69	STEMI	Multi-vessel PCI could increase the duration of the procedure and the contrast load. However, multi-vessel PCI did not increase the in-hospital MACEs.	[28]
Non-randomized studies					
Cavender *et al*.	2009		STEMI	Multi-vessel PCI increased the risk of in-hospital mortality, including in patients with cardiogenic shock.	[15]
Hannan *et al*.	2010		STEMI	Immediate complete revascularization increased the incidence of in-hospital mortality, excluding the cardiogenic shock patients. However, staged complete revascularization decreased this risk within one year.	[12]
Toma *et al*.	2010		STEMI	Complete revascularization increased the risk of death within 90-day.	[16]
Kong *et al*.	2006		AMI	Complete revascularization decreased the risk of in-hospital death.	[17]
Chen *et al*.	2005		AMI	No differences were found.	[18]
Corpus *et al*.	2004		AMI	Multi-vessel PCI increased the risk of re-infarction revascularization and major adverse cardiac events at one year.	[19]
Varani *et al*.	2008		STEMI	Multi-vessel PCI decreased risk of mortality at one month, excluding the patients with cardiogenic shock.	[20]
Roe *et al*.	2001		AMI	Multi-vessel PCI did not increase the risk of death, re-infarction, and stroke at 6 months.	[21]
Rigattieri *et al*.	2008		STEMI	Multi-vessel PCI did not significantly increase the risk of major adverse cardiac events in hospital, yet decreased the risk of major adverse cardiac events out of hospital.	[22]
Khattab *et al*.	2008		AMI	No differences were found.	[23]
Mohamad *et al*.	2011		STEMI	No differences were found.	[24]

STEMI, ST-segment elevation myocardial infarction; MACEs, major adverse cardiac events; MVD, multivessel disease; PCI, percutaneous coronary intervention; HELP AMI, HEpacoat for cuLPrit or multivessel stenting for Acute Myocardial Infarction; COR, culprit-only revascularization; AMI, acute myocardial infarction.

The COMPLETE trial is a large-sample, multicenter, randomized controlled trial designed to investigate the superiority of complete revascularization over COR. A total of 4041 patients were enrolled in this study, which demonstrated that complete revascularization could significantly reduce the incidence of cardiovascular death or myocardial infarction (HR 0.74, CI: 0.60–0.91, *p* = 0.004), ischemia-driven revascularization (HR 0.18, CI: 0.12–0.26) and unstable angina (HR 0.53, CI: 0.40–0.71). However, no significant distinctions were observed in major bleeding between those two groups (HR 1.33, CI: 0.90–1.97, *p* = 0.15) [4]. CvLPRIT is an open-label randomized trial involving 296 patients that compared complete revascularization with only the revascularization of the infarct-related artery. This study performed complete revascularization either during primary PCI or before hospital discharge. Although complete revascularization significantly reduced the primary outcome (HR 0.45, CI: 0.24–0.84, *p* = 0.009), a composite outcome of all-cause death, recurrent myocardial infarction, heart failure, and ischemia-driven revascularization, it did not reduce death (HR 0.38, CI: 0.12–1.20, *p* = 0.09) and recurrent myocardial infarction (HR 0.47, CI: 0.09–2.59, *p* = 0.38) [25].

A meta-analysis conducted by Elgendy *et al*. [29] aimed to examine the efficacy and safety of complete revascularization in patients with MVD. This study included a total of 10 trials involving 2285 patients. The findings indicated that complete revascularization was associated with a lower risk of MACEs (RR 0.57, CI: 0.42–0.77), primarily attributed to the risk of urgent revascularization reduced at 56% (RR 0.44, CI: 0.30–0.66). Furthermore, no significant distinction was observed between groups receiving complete or incomplete revascularization procedures regarding all-cause death (RR 0.76, CI: 0.52–1.12) and re-infarction (RR 0.54, CI: 0.23–1.27). Importantly, the reduction in the risk of MACEs remained consistent regardless of the timing for non-culprit artery revascularization, as indicated by the mixed treatment model.

## 3. Can The Application of FFR Bring More Net Clinical Benefits for Complete Revascularization?

Intracavitary imaging and functional assessment are practical tools for accurately assessing the extent of stenosis and guiding interventional therapy. These are widely used to assess critical lesions in patients with stable coronary artery disease [13]. However, it is unclear whether applying FFR can offer increased net clinical benefits for patients with STEMI and MVD. Five randomized controlled trials have already explored the application of FFR in complete revascularization [6, 7, 30, 31, 32].

The DANAMI-3-PRIMULTI trial was an open-label, randomized, controlled study comparing FFR-guided revascularization with COR. The results suggested that the FFR-guided PCI decreased the risk of ischemia-driven revascularization to 69% (HR 0.31, CI: 0.18–0.53, *p* < 0.0001). However, no differences were found in all-cause death (*p* = 0.43) and non-fatal re-infarction (*p* = 0.87) [6]. Neupane *et al*. [33] conducted a meta-analysis to compare FFR-guided complete revascularization with COR, which included three trials and 1633 patients. This study suggested that FFR-guided PCI was associated with a lower incidence of MACEs than COR in patients with STEMI and MVD. This distinction was driven by a lower rate of repeat revascularization in the FFR-guided group [33].

The FRAME-AMI trial was conducted in Korea at 14 sites and involved 562 patients. This study compared the outcomes of FFR-guided complete revascularization with angiography-guided complete revascularization. In the group that underwent FFR-guided complete revascularization, 60% of patients received immediate PCI, while 40% underwent a staged procedure during their hospital stay. The findings revealed that the FFR-guided group had fewer stents applied (*p* < 0.001). Furthermore, it was observed that the FFR-guided PCI group experienced a significant reduction in the incidence of primary endpoints (including death, myocardial infarction, and repeat revascularization) compared to the angiography-guided PCI group (HR 0.43, CI: 0.25–0.75, *p* = 0.003). Additionally, there was also a lower occurrence of death (HR 0.30, CI: 0.11–0.83, *p* = 0.020) and myocardial infarction (HR 0.32, CI: 0.13–0.75, *p* = 0.009) [31]. However, contrasting results were obtained from the FLOWER-MI trial, which demonstrated no significant difference between the FFR-guided group and angiography-guided group regarding primary outcome measures (HR 1.32, CI: 0.78–2.23), death (HR 0.89, CI: 0.36–2.20), or non-fatal myocardial infarction (HR 1.77, CI: 0.82–3.84) [32]. It is worth noting that during acute stages, FFR may lead to overestimating stenosis severity in non-infarct arteries, potentially explaining these conflicting findings between the trials.

Currently, the decision to perform revascularization is based primarily on the degree of stenosis, although the plaque instability may not be limited to the degree of stenosis. However, vasospasm may cause false positives following the application of FFR.

## 4. When is the Optimal Timing for Complete Revascularization? 

**Fig. 2.**
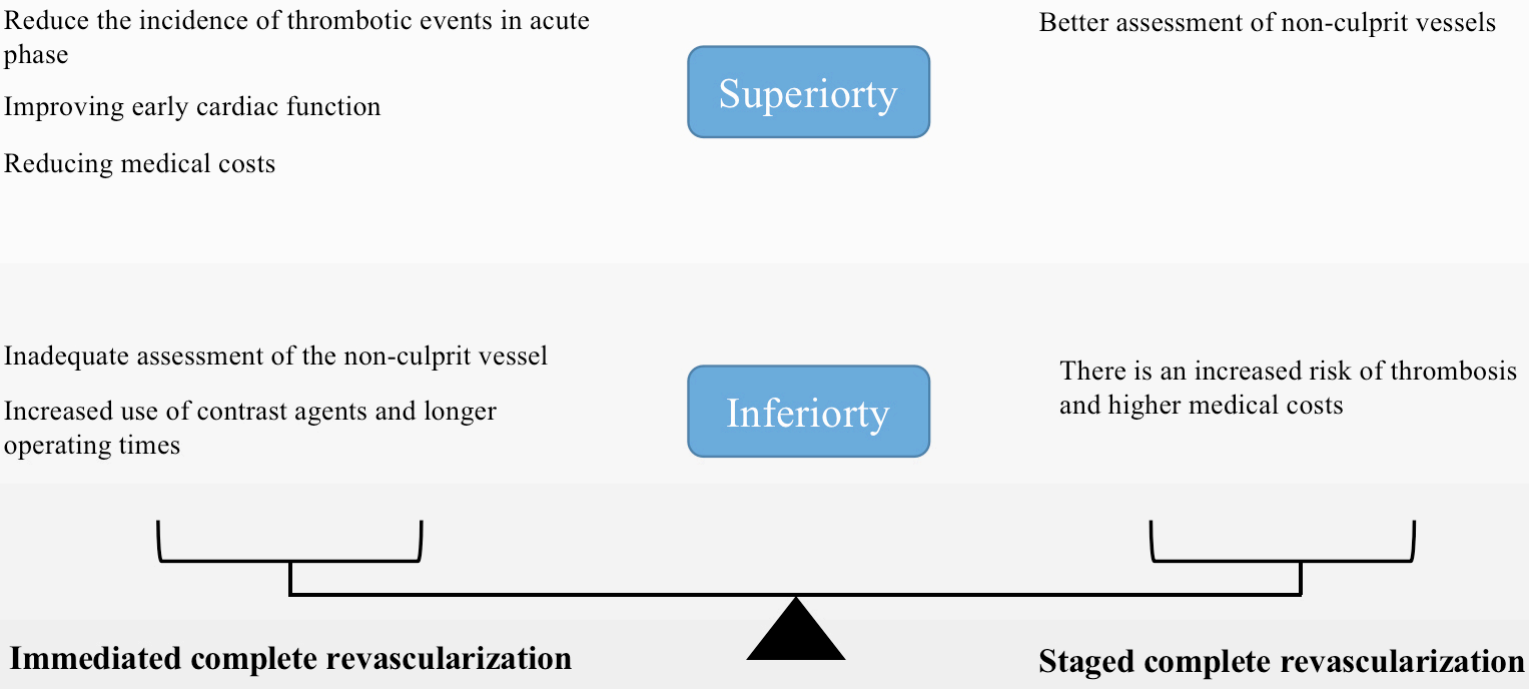
**The superiorities and inferiorities of two different methods of complete revascularization**.

Complete revascularization is superior to COR in patients with acute coronary syndrome (ACS) and MVD. However, whether immediate complete revascularization is associated with more superiority than staged complete revascularization is unclear. The superiority and inferiority of immediate and staged complete revascularization are shown (Fig. 2). About eight randomized controlled trials have explored this topic, and BIOVASC was the first large-scale randomized controlled trial (Table 2, Ref. [34, 35, 36, 37, 38, 39]).

**Table 2.** **The published studies comparing immediate complete revascularization with staged complete revascularization**.

Trials	Sample	Year	Patients	Conclusions	Reference
Politi *et al*.	214	2010	STEMI	Staged revascularization is similar to immediate complete revascularization in the incidence of MACEs.	[34]
Maamoun *et al*.	78	2011	STEMI	Immediate multi-vessel strategy PCI is safe and feasible with no clear long-term benefits to MACE rates over the staged multi-vessel strategy.	[35]
Tarasov *et al*.	227	2013	STEMI	Immediate complete revascularization and the secondary revascularization demonstrated comparable results for 12 months.	[36]
Sardell *et al*.	527	2016	Non-STEMI	Immediate complete revascularization is superior to staged revascularization in terms of major adverse cardiovascular and cerebrovascular events.	[37]
Diletti *et al*.	1525	2023	ACS	Immediate complete revascularization was similar to staged complete revascularization.	[38]
Stähli *et al*.	840	2023	STEMI	Immediate multivessel PCI was similar to staged multivessel PCI.	[39]

STEMI, ST-segment elevation myocardial infarction; MACEs, major adverse cardiac events; PCI, percutaneous coronary intervention; ACS, acute coronary syndrome.

The BIOVASC trial was a randomized trial conducted in 29 hospitals across Belgium, Italy, the Netherlands, and Spain. It aimed to compare the outcomes of immediate and staged complete revascularization in a large sample group. A total of 764 patients were assigned to the immediate group, while 761 patients were assigned to the staged group. The findings indicated that immediate revascularization was similar to staged revascularization at one year (7.6% vs. 9.4%, HR 0.78, CI: 0.55–1.11, P _non-inferiority_ = 0.0011). Moreover, immediate complete revascularization demonstrated additional benefits by reducing the incidence of myocardial infarction (HR 0.41, CI: 0.22–0.76, *p* = 0.0045) and unplanned ischemia-driven revascularizations (HR 0.61, CI: 0.39–0.95, *p* = 0.030). However, it did not significantly impact all-cause mortality rates (HR 1.56, CI: 0.68–3.61, *p* = 0.30) [38]. Another similar study called MULTISTARS AMI also reached comparable conclusions during its presentation at the ESC Congress in 2023. This study suggested that among hemodynamically stable STEMI patients with MVD, immediate multivessel PCI was equally effective as staged multivessel PCI in terms of risk reduction for death from any-cause, fatal or non-fatal myocardial infarction, strokes, and hospitalizations due to heart failure within one year [39].

Agarwal *et al*. [40] conducted a meta-analysis that included six randomized controlled trials to evaluate the efficacy and safety of immediate complete revascularization. The results suggested that immediate complete revascularization increased the risk of myocardial infarction or death (RR 1.66, CI: 1.09–2.52, *p* = 0.02) and all-cause mortality (RR 2.55, CI: 1.42–4.58, *p* < 0.01) by 66% and 155%, respectively. At the same time, immediate complete revascularization could increase the incidence of cardiovascular mortality (RR 2.8, CI: 1.33–5.86, *p* = 0.01) compared to staged complete revascularization. On the contrary, no distinctions were found in MACEs (RR 1.14, CI: 0.88–1.49, *p* = 0.33), repeat revascularization (RR 0.92, CI: 0.66–1.28, *p* = 0.62), and repeat myocardial infarction (RR 1.14, CI: 0.68–1.92, *p* = 0.61). However, this meta-analysis only included six trials and 1126 patients, which lacked sufficient evidence from large clinical research studies.

Cui *et al*. [41] performed a network meta-analysis to research the optional strategies for patients with MVD. This study included 6942 patients and compared immediate, staged, and COR. Compared with COR, immediate and staged complete revascularization were associated with 52% and 27% reductions in the risk of cardiovascular death (RR 0.48, CI: 0.32–0.73, $I^{2}$ = 0%) and MI (RR 0.73, CI: 0.61–0.88, $I^{2}$ = 0%), respectively.

## 5. Does Complete Revascularization Bring More Clinical Benefits to Patients with MVD and Cardiogenic Shock?

Cardiogenic shock is a fatal and common complication after acute myocardial infarction with an incidence of about 6–12%, which significantly increases the risk of mortality [42]. A previous randomized study suggested that early revascularization in patients with acute myocardial infarction and cardiogenic shock could improve short-term and long-term survival rates [43]. However, despite the increasing application of early revascularization with PCI among these patients, a high mortality rate of 40–50% remained [44]. Therefore, immediate complete revascularization was regarded as a contraindication in patients with STEMI and cardiogenic shock.

The CULPRIT-SHOCK [45] trial is a unique randomized controlled study that compares two initial revascularization strategies in patients with STEMI and cardiogenic shock. A total of 344 patients were assigned to undergo culprit lesion-only revascularization, while the other group received immediate multivessel PCI. The findings reveal that culprit-lesion-only revascularization significantly reduces the combined primary outcome of death or renal-replacement therapy (RR 0.83, CI: 0.71–0.96, *p* = 0.01) compared to immediate complete revascularization. Furthermore, the culprit-lesion-only PCI group exhibited a lower relative risk of death than the multivessel PCI group (RR 0.84, CI: 0.72–0.98, *p* = 0.03). Moreover, it demonstrated a trend toward the reduced need for renal replacement therapy (RR 0.71, CI: 0.49–1.03, *p* = 0.07). Notably, no significant distinctions were observed between these two groups regarding the time taken for hemodynamic stabilization, the requirement for catecholamine therapy and its duration, levels of troponin T and creatine kinase, and rates of bleeding and stroke. Furthermore, Xiong *et al*. [44] performed an updated meta-analysis including fifteen studies comparing clinical outcomes between multivessel PCI and culprit vessel-only PCI, which showed that immediate complete revascularization does not provide significant benefits for patients with STEMI complicating cardiogenic shock. At the same time, multivessel PCI increased the risk of developing acute renal failure compared to culprit vessel-only PCI (OR = 1.33, CI: 1.04–1.69).

Although performing immediate complete revascularization in patients with cardiogenic shock presents significant challenges and may increase the risk of patient death, this could change following the application of venoarterial-extracorporeal membrane oxygenation. However, it remains unclear whether mechanical circulatory support devices can reduce the risk of death and improve cardiac function in the early stages. A study conducted by Choi *et al*. [46] indicated that immediate multivessel PCI plus venoarterial-extracorporeal membrane oxygenation was linked to a lower risk of 30-day mortality or renal-replacement therapy (68% vs. 54.3%; *p* = 0.018) as well as all-cause mortality (HR 0.689, CI: 0.506–0.939), compared with COR, in patients with STEMI complicating advanced cardiogenic shock. However, the ECSL-SHOCK trial showed that extracorporeal life support did not offer improved clinical benefits for STEMI patients with cardiogenic shock undergoing revascularization than medical treatment alone [47]. Hence, there is a need for more randomized controlled trials to explore the efficacy of extracorporeal life support in those patients.

## 6. Surgical Revascularization for Patients with STEMI

Coronary artery bypass grafting (CABG) is one of the best strategies for patients with MVD, especially in diabetic patients. However, CABG is rarely performed in the acute phase of patients with STEMI, and the optimal timing of CABG operations in patients with recent acute myocardial infarction is unclear. Therefore, evidence is insufficient to compare the different surgical revascularization techniques in patients with STEMI. Fattouch *et al*. [48] conducted a study comprised of on-pump with off-pump CABG surgery in patients with STEMI. This study suggested that off-pump CABG was associated with similar late mortality, MACEs, and graft patency rates compared to conventional cardioplegic cardiac arrest CABG.

## 7. What do the Current Guidelines Recommend?

The guidelines for managing acute coronary syndromes were updated by the European Society of Cardiology in 2023. It is recommended to perform culprit vessel revascularization (Ia) during primary PCI, followed by staged complete revascularization (IIa) for patients with MVD and cardiogenic shock. Furthermore, STEMI patients without cardiogenic shock should undergo complete revascularization either during the initial procedure or within 45 days. Moreover, immediate complete revascularization should be considered in patients with non-ST-segment elevation acute coronary syndrome (NSTE-ACS) and MVD. Lastly, PCI for non-infarct-related arteries should be based on the severity of the angiographic findings [49].

## 8. Conclusions

(1) Complete revascularization is superior to COR in patients with STEMI and MVD, as evidenced by a series of randomized trials and recommended by clinical guidelines as Class Ia.

(2) Based on current evidence, FFR-guided complete revascularization is inferior to anatomic complete revascularization in patients with STEMI.

(3) Increasingly, large-scale clinical trials favor immediate complete revascularization in patients without cardiogenic shock.

(4) COR is the best choice for patients with STEMI and cardiogenic shock. However, applying ECMO could enhance the superiority of immediate complete revascularization, and we expect more randomized controlled trials to provide evidence on these topics.

## References

[1] Martí D, Mestre JL, Salido L, Esteban MJ, Casas E, Pey J (2014). Incidence, angiographic features and outcomes of patients presenting with subtle ST-elevation myocardial infarction. *American Heart Journal*.

[2] Park DW, Clare RM, Schulte PJ, Pieper KS, Shaw LK, Califf RM (2014). Extent, location, and clinical significance of non-infarct-related coronary artery disease among patients with ST-elevation myocardial infarction. *JAMA*.

[3] Sorajja P, Gersh BJ, Cox DA, McLaughlin MG, Zimetbaum P, Costantini C (2007). Impact of multivessel disease on reperfusion success and clinical outcomes in patients undergoing primary percutaneous coronary intervention for acute myocardial infarction. *European Heart Journal*.

[4] Mehta SR, Wood DA, Storey RF, Mehran R, Bainey KR, Nguyen H (2019). Complete Revascularization with Multivessel PCI for Myocardial Infarction. *The New England Journal of Medicine*.

[5] Wald DS, Morris JK, Wald NJ, Chase AJ, Edwards RJ, Hughes LO (2013). Randomized trial of preventive angioplasty in myocardial infarction. *The New England Journal of Medicine*.

[6] Engstrøm T, Kelbæk H, Helqvist S, Høfsten DE, Kløvgaard L, Holmvang L (2015). Complete revascularisation versus treatment of the culprit lesion only in patients with ST-segment elevation myocardial infarction and multivessel disease (DANAMI-3—PRIMULTI): an open-label, randomised controlled trial. *Lancet (London, England)*.

[7] Smits PC, Abdel-Wahab M, Neumann FJ, Boxma-de Klerk BM, Lunde K, Schotborgh CE (2017). Fractional Flow Reserve-Guided Multivessel Angioplasty in Myocardial Infarction. *The New England Journal of Medicine*.

[8] Gershlick AH, Banning AS, Parker E, Wang D, Budgeon CA, Kelly DJ (2019). Long-Term Follow-Up of Complete Versus Lesion-Only Revascularization in STEMI and Multivessel Disease: The CvLPRIT Trial. *Journal of the American College of Cardiology*.

[9] Windecker S, Kolh P, Alfonso F, Collet JP, Cremer J, Authors/Task Force members (2014). 2014 ESC/EACTS Guidelines on myocardial revascularization: The Task Force on Myocardial Revascularization of the European Society of Cardiology (ESC) and the European Association for Cardio-Thoracic Surgery (EACTS) Developed with the special contribution of the European Association of Percutaneous Cardiovascular Interventions (EAPCI). *European Heart Journal*.

[10] O’Gara PT, Kushner FG, Ascheim DD, Casey DE, Chung MK, de Lemos JA (2013). 2013 ACCF/AHA guideline for the management of ST-elevation myocardial infarction: a report of the American College of Cardiology Foundation/American Heart Association Task Force on Practice Guidelines. *Circulation*.

[11] Kornowski R, Mehran R, Dangas G, Nikolsky E, Assali A, Claessen BE (2011). Prognostic impact of staged versus ”one-time” multivessel percutaneous intervention in acute myocardial infarction: analysis from the HORIZONS-AMI (harmonizing outcomes with revascularization and stents in acute myocardial infarction) trial. *Journal of the American College of Cardiology*.

[12] Hannan EL, Samadashvili Z, Walford G, Holmes DR, Jacobs AK, Stamato NJ (2010). Culprit vessel percutaneous coronary intervention versus multivessel and staged percutaneous coronary intervention for ST-segment elevation myocardial infarction patients with multivessel disease. *JACC. Cardiovascular Interventions*.

[13] Matsuo H, Kawase Y (2016). FFR and iFR guided percutaneous coronary intervention. *Cardiovascular Intervention and Therapeutics*.

[14] Gaba P, Gersh BJ, Ali ZA, Moses JW, Stone GW (2021). Complete versus incomplete coronary revascularization: definitions, assessment and outcomes. *Nature Reviews. Cardiology*.

[15] Cavender MA, Milford-Beland S, Roe MT, Peterson ED, Weintraub WS, Rao SV (2009). Prevalence, predictors, and in-hospital outcomes of non-infarct artery intervention during primary percutaneous coronary intervention for ST-segment elevation myocardial infarction (from the National Cardiovascular Data Registry). *The American Journal of Cardiology*.

[16] Toma M, Buller CE, Westerhout CM, Fu Y, O’Neill WW, Holmes DR (2010). Non-culprit coronary artery percutaneous coronary intervention during acute ST-segment elevation myocardial infarction: insights from the APEX-AMI trial. *European Heart Journal*.

[17] Kong JA, Chou ET, Minutello RM, Wong SC, Hong MK (2006). Safety of single versus multi-vessel angioplasty for patients with acute myocardial infarction and multi-vessel coronary artery disease: report from the New York State Angioplasty Registry. *Coronary Artery Disease*.

[18] Chen LY, Lennon RJ, Grantham JA, Berger PB, Mathew V, Singh M (2005). In-hospital and long-term outcomes of multivessel percutaneous coronary revascularization after acute myocardial infarction. *The American Journal of Cardiology*.

[19] Corpus RA, House JA, Marso SP, Grantham JA, Huber KC, Laster SB (2004). Multivessel percutaneous coronary intervention in patients with multivessel disease and acute myocardial infarction. *American Heart Journal*.

[20] Varani E, Balducelli M, Aquilina M, Vecchi G, Hussien MN, Frassineti V (2008). Single or multivessel percutaneous coronary intervention in ST-elevation myocardial infarction patients. *Catheterization and Cardiovascular Interventions: Official Journal of the Society for Cardiac Angiography & Interventions*.

[21] Roe MT, Cura FA, Joski PS, Garcia E, Guetta V, Kereiakes DJ (2001). Initial experience with multivessel percutaneous coronary intervention during mechanical reperfusion for acute myocardial infarction. *The American Journal of Cardiology*.

[22] Rigattieri S, Biondi-Zoccai G, Silvestri P, Di Russo C, Musto C, Ferraiuolo G (2008). Management of multivessel coronary disease after ST elevation myocardial infarction treated by primary angioplasty. *Journal of Interventional Cardiology*.

[23] Khattab AA, Abdel-Wahab M, Röther C, Liska B, Toelg R, Kassner G (2008). Multi-vessel stenting during primary percutaneous coronary intervention for acute myocardial infarction. A single-center experience. *Clinical Research in Cardiology: Official Journal of the German Cardiac Society*.

[24] Mohamad T, Bernal JM, Kondur A, Hari P, Nelson K, Niraj A (2011). Coronary revascularization strategy for ST elevation myocardial infarction with multivessel disease: experience and results at 1-year follow-up. *American Journal of Therapeutics*.

[25] Gershlick AH, Khan JN, Kelly DJ, Greenwood JP, Sasikaran T, Curzen N (2015). Randomized trial of complete versus lesion-only revascularization in patients undergoing primary percutaneous coronary intervention for STEMI and multivessel disease: the CvLPRIT trial. *Journal of the American College of Cardiology*.

[26] Dambrink JHE, Debrauwere JP, van ‘t Hof AWJ, Ottervanger JP, Gosselink ATM, Hoorntje JCA (2010). Non-culprit lesions detected during primary PCI: treat invasively or follow the guidelines. *EuroIntervention: Journal of EuroPCR in Collaboration with the Working Group on Interventional Cardiology of the European Society of Cardiology*.

[27] Hamza M, Mahmoud A, Elgendy IY (2016). A Randomized Trial of Complete Versus Culprit-Only Revascularization During Primary Percutaneous Coronary Intervention in Diabetic Patients With Acute ST Elevation Myocardial Infarction and Multi Vessel Disease. *Journal of Interventional Cardiology*.

[28] Di Mario C, Mara S, Flavio A, Imad S, Antonio M, Anna P (2004). Single vs multivessel treatment during primary angioplasty: results of the multicentre randomised HEpacoat for cuLPrit or multivessel stenting for Acute Myocardial Infarction (HELP AMI) Study. *International Journal of Cardiovascular Interventions*.

[29] Elgendy IY, Mahmoud AN, Kumbhani DJ, Bhatt DL, Bavry AA (2017). Complete or Culprit-Only Revascularization for Patients With Multivessel Coronary Artery Disease Undergoing Percutaneous Coronary Intervention: A Pairwise and Network Meta-Analysis of Randomized Trials. *JACC. Cardiovascular Interventions*.

[30] Ghani A, Dambrink JHE, van ‘t Hof AWJ, Ottervanger JP, Gosselink ATM, Hoorntje JCA (2012). Treatment of non-culprit lesions detected during primary PCI: long-term follow-up of a randomised clinical trial. *Netherlands Heart Journal: Monthly Journal of the Netherlands Society of Cardiology and the Netherlands Heart Foundation*.

[31] Lee JM, Kim HK, Park KH, Choo EH, Kim CJ, Lee SH (2023). Fractional flow reserve versus angiography-guided strategy in acute myocardial infarction with multivessel disease: a randomized trial. *European Heart Journal*.

[32] Puymirat E, Cayla G, Simon T, Steg PG, Montalescot G, Durand-Zaleski I (2021). Multivessel PCI Guided by FFR or Angiography for Myocardial Infarction. *New England Journal of Medicine*.

[33] Neupane S, Singh H, Edla S, Altujjar M, Yamsaki H, Lalonde T (2019). Meta-analysis of fractional flow reserve guided complete revascularization versus infarct related artery only revascularization in patients with ST-elevation myocardial infarction and multivessel coronary artery disease. *Coronary Artery Disease*.

[34] Politi L, Sgura F, Rossi R, Monopoli D, Guerri E, Leuzzi C (2010). A randomised trial of target-vessel versus multi-vessel revascularisation in ST-elevation myocardial infarction: major adverse cardiac events during long-term follow-up. *Heart (British Cardiac Society)*.

[35] Maamoun W, Elkhaeat N, Elarasy R (2011). Safety and feasibility of complete simultaneous revascularization during primary PCI in patients with STEMI and multi-vessel disease. *The Egyptian Heart Journal*.

[36] Tarasov RS, Ganiukov VI, Popov VA, Shushpannikov PA, Barbarash OL, Barbarash LS (2013). Effect of the terms of complete revascularization on the outcomes of treatment of patients with st segment elevation myocardial infarction and multivessel coronary artery disease. *Angiologiia i Sosudistaia Khirurgiia = Angiology and Vascular Surgery*.

[37] Sardella G, Lucisano L, Garbo R, Pennacchi M, Cavallo E, Stio RE (2016). Single-Staged Compared With Multi-Staged PCI in Multivessel NSTEMI Patients: The SMILE Trial. *Journal of the American College of Cardiology*.

[38] Diletti R, den Dekker WK, Bennett J, Schotborgh CE, van der Schaaf R, Sabaté M (2023). Immediate versus staged complete revascularisation in patients presenting with acute coronary syndrome and multivessel coronary disease (BIOVASC): a prospective, open-label, non-inferiority, randomised trial. *Lancet (London, England)*.

[39] Stähli BE, Varbella F, Linke A, Schwarz B, Felix SB, Seiffert M (2023). Timing of Complete Revascularization with Multivessel PCI for Myocardial Infarction. *The New England Journal of Medicine*.

[40] Agarwal N, Jain A, Garg J, Mojadidi MK, Mahmoud AN, Patel NK (2017). Staged versus index procedure complete revascularization in ST-elevation myocardial infarction: A meta-analysis. *Journal of Interventional Cardiology*.

[41] Cui K, Yin D, Zhu C, Yuan S, Wu S, Feng L (2022). Optimal Revascularization Strategy for Patients With ST-segment Elevation Myocardial Infarction and Multivessel Disease: A Pairwise and Network Meta-Analysis. *Frontiers in Cardiovascular Medicine*.

[42] Zeymer U, Hochadel M, Thiele H, Andresen D, Schühlen H, Brachmann J (2015). Immediate multivessel percutaneous coronary intervention versus culprit lesion intervention in patients with acute myocardial infarction complicated by cardiogenic shock: results of the ALKK-PCI registry. *EuroIntervention: Journal of EuroPCR in Collaboration with the Working Group on Interventional Cardiology of the European Society of Cardiology*.

[43] Hochman JS, Sleeper LA, Webb JG, Dzavik V, Buller CE, Aylward P (2006). Early revascularization and long-term survival in cardiogenic shock complicating acute myocardial infarction. *JAMA*.

[44] Xiong B, Yang H, Yu W, Zeng Y, Han Y, She Q (2022). Multivessel vs. Culprit Vessel-Only Percutaneous Coronary Intervention for ST-Segment Elevation Myocardial Infarction in Patients With Cardiogenic Shock: An Updated Systematic Review and Meta-Analysis. *Frontiers in Cardiovascular Medicine*.

[45] Thiele H, Akin I, Sandri M, Fuernau G, de Waha S, Meyer-Saraei R (2017). PCI Strategies in Patients with Acute Myocardial Infarction and Cardiogenic Shock. *The New England Journal of Medicine*.

[46] Choi KH, Yang JH, Park TK, Lee JM, Song YB, Hahn JY (2023). Culprit-Only Versus Immediate Multivessel Percutaneous Coronary Intervention in Patients With Acute Myocardial Infarction Complicating Advanced Cardiogenic Shock Requiring Venoarterial-Extracorporeal Membrane Oxygenation. *Journal of the American Heart Association*.

[47] Thiele H, Zeymer U, Akin I, Behnes M, Rassaf T, Mahabadi AA (2023). Extracorporeal Life Support in Infarct-Related Cardiogenic Shock. *The New England Journal of Medicine*.

[48] Fattouch K, Runza G, Moscarelli M, Trumello C, Incalcatera E, Corrado E (2011). Graft patency and late outcomes for patients with ST-segment elevation myocardial infarction who underwent coronary surgery. *Perfusion*.

[49] Byrne RA, Rossello X, Coughlan JJ, Barbato E, Berry C, Chieffo A (2023). 2023 ESC Guidelines for the management of acute coronary syndromes. *European Heart Journal*.

